# A New Model of Delirium Care in the Acute Geriatric Setting: Geriatric Monitoring Unit

**DOI:** 10.1186/1471-2318-11-41

**Published:** 2011-08-13

**Authors:** Mei Sian Chong, Mark PC Chan, Jasmine Kang, Huey Charn Han, Yew Yoong Ding, Thai Lian Tan

**Affiliations:** 1 Department of Geriatric Medicine. Tan Tock Seng Hospital. 11 Jalan Tan Tock Seng. Singapore; 2 Nursing Service. Tan Tock Seng Hospital. 11 Jalan Tan Tock Seng. Singapore

## Abstract

**Background:**

Delirium is a common and serious condition, which affects many of our older hospitalised patients. It is an indicator of severe underlying illness and requires early diagnosis and prompt treatment, associated with poor survival, functional outcomes with increased risk of institutionalisation following the delirium episode in the acute care setting. We describe a new model of delirium care in the acute care setting, titled Geriatric Monitoring Unit (GMU) where the important concepts of delirium prevention and management are integrated. We hypothesize that patients with delirium admitted to the GMU would have better clinical outcomes with less need for physical and psychotropic restraints compared to usual care.

**Methods/Design:**

GMU models after the Delirium Room with adoption of core interventions from Hospital Elder Life Program and use of evening bright light therapy to consolidate circadian rhythm and improve sleep in the elderly patients. The novelty of this approach lies in the amalgamation of these interventions in a multi-faceted approach in acute delirium management. GMU development thus consists of key considerations for room design and resource planning, program specific interventions and daily core interventions. Assessments undertaken include baseline demographics, comorbidity scoring, duration and severity of delirium, cognitive, functional measures at baseline, 6 months and 12 months later. Additionally we also analysed the pre and post-GMU implementation knowledge and attitude on delirium care among staff members in the geriatric wards (nurses, doctors) and undertook satisfaction surveys for caregivers of patients treated in GMU.

**Discussion:**

This study protocol describes the conceptualization and implementation of a specialized unit for delirium management. We hypothesize that such a model of care will not only result in better clinical outcomes for the elderly patient with delirium compared to usual geriatric care, but also improved staff knowledge and satisfaction. The model may then be transposed across various locations and disciplines in the acute hospital where delirious patients could be sited.

**Trial Registration:**

Current Controlled Trials ISRCTN52323811

## Background

Delirium is a common and serious condition, which affects many of our older hospitalised patients. The prevalence in hospitalised elderly patients is shown to be as high as 50%, with the diagnosis in 11-24% of older patients upon admission and another 5-35% of them developing delirium during admission [1,2]. It is an indicator of severe underlying illness and requires early diagnosis and prompt treatment. The development of delirium is associated with increased need for nursing surveillance, greater hospital costs and in-hospital mortality rates of 25-33% and one-year mortality rate of 35-40% [3-8]. Complications may also occur related to physical injuries from agitated behaviour, complications of immobility as a result of physical restraints and pharmacologic agents used to control the behaviour in the confused patients and nosocomial infections resulting from the prolonged hospitalization [9]. This in turn translates to increases in length of hospital stay, rates of admission to nursing homes, rates of mortality, and higher healthcare costs.

The importance of delirium is highlighted by its inclusion as a marker for quality of care and patient safety by the National Quality Measures Clearinghouse of the Agency for Healthcare Research and Quality, as higher delirium rates would be expected to correlate with lower quality of hospital care after adjustment for casemix. Moreover, the Assessing Care of Vulnerable Elders Project has ranked delirium among the top 3 conditions for which the quality of care needs to be improved [10,11]. Other worldwide initiatives such as in Western Australia (WA), the Department of Health will be implementing evidenced based Models of Care (including Delirium) across WA Health with other nation-wide efforts made in UK as well.

The Asian state of Singapore has one of the fastest ageing populations in the Asia-Pacific region with 15-20% of total population consisting of persons aged 65 and above by the year 2030. A random point survey in our geriatric medicine department demonstrated a delirium prevalence rate of 15%. This potentially translates to 3,600 patients per year or 10 patients per day. The survey also showed that elderly inpatients with delirium had a longer average length of stay (ALOS)(15 days) than that of a normal geriatric inpatient (ALOS = 11 days). In addition, 50% of falls in the geriatric wards were related to delirium, resulting in an even longer ALOS of 23.9 days, thus strengthening the case for improved delirium care (data available upon request).

Studies have looked at interventions for delirium prevention in hospitalised geriatric patients [12], yet others have incorporated delirium management as part of the interventions of a consulting geriatric team [13-16] or nursing improvement protocols [17-19]. However few studies have specifically examined the impact of combined measures to improve the management of subjects with delirium (Table 1) [20-25]. The mixed results illustrated serve to highlight the heterogeneity of interventions employed and outcomes measured. Despite various differences, a common theme of these programs appears to be the application of comprehensive medical care with emphasis on intensive nursing care and education.

**Table 1:** Summary of studies on delirium management in acute hospitals

Study	Subjects, n	Interventions	Outcomes	Results
Cole et al, 1994	88	• Geriatric consultation within 24 hours • Daily nursing visits • Nursing intervention protocol	• SPMSQ (memory, orientation, concentration) • CAM • CGBRS (behaviour and ADLs)	• Improvement in SPMSQ scores at 2 weeks but no difference at 8 weeks • No difference in restraint use, LOS, discharge setting, survival
Cole et al, 2002	227	• Geriatrician consult within 24 hours • Daily nurse visits • Nursing intervention protocol	• MMSE • Delirium Index • Barthel index • LOS • Survival within 8 weeks • Charlson's	• No difference in time to improvement • No difference in delirium index, Barthel, LOS, discharge setting, and survival.
Flaherty et al, 2003	196, 69 with delirium	• 24 hour intensive nursing care • Physical restraints-free policy • Comprehensive geriatric care	• Not applicable (descriptive study)	• No physical restraints used during patient hospital stay. • Lower use of pharmacological restraints • LOS equal to expected LOS
Lundstrom et al, 2005	400, 125 with delirium	• 2-day education course for staff on Geriatrics • Education for caregiver-patient interaction • Work reorganization (focus on individualized care) • Monthly staff guidance	• OBS scale • MMSE • Katz ADL • DSM IV for delirium	• Fewer patients remained delirious on day 7. • LOS lower with intervention. • Fewer deaths in subjects in intervention ward.
Pitkala et al, 2006, 2008	174	• Comprehensive geriatric assessment • Administering atypical antipsychotics instead of conventional neuroleptics • Orientation • Physiotherapy • Nutritional supplementation • Hip protectors • Cholinesterase inhibitors • Discharge planning	• Discharge destination and mortality • MMSE • Barthel • MNA • Geriatric Depression Scale • MDAS • Costs & HRQoL	• No significant difference in primary outcome (Institutionalisation and mortality) • Intensity and symptoms of delirium were alleviated faster in treatment group • Improvement in cognition at 6 months • No difference in change of function

ADL: activities of daily living; CAM: confusion assessment method; CGBRS: Crichton geriatric behavioural rating scale; DSM-IV: diagnostic and statistical manual of mental disorders, fourth edition; HRQoL: health-related quality of life; LOS: length of stay; MDAS: memorial delirium assessment scale; MMSE:mini-mental state examination; MNA: mini nutritional assessment; OBS: organic brain syndrome scale; SPMSQ: short portable mental status questionnaire.

In improving delirium care in the acute hospitalised elderly, the establishment of a specific unit for acute delirium care represents a significant step towards the systematic delivery of good geriatric care for the delirious elderly. For the current study, we describe the development of the Geriatric Monitoring Unit (GMU)- a specialised 5-bedded unit which incorporates specific room design for an elder-friendly environment, lower staff-patient ratio, coupled with structured core, program interventions and bright light therapy to reside delirious patients, supported by 24-hour intensive nursing care.

The primary aim of this study is to compare delirious patients admitted to the Geriatric Monitoring Unit (GMU), compared to usual geriatric care in general ward setting on the following primary measures: (1) Duration of delirium and severity of delirium, (2) Use of physical and chemical restraints, (3) Falls rate, (4) Physical injury rate due to agitated behavior, (5) Functional outcomes/gains in the elderly geriatric patients, (6) Morbidity (such as nosocomial infection rate, urinary catheter use and catheter days, pressure ulcer rate); (7) Patient and family satisfaction score in elderly persons with delirium. We postulate better outcomes in patients admitted to GMU versus usual geriatric care. Our secondary aims include looking at which specific aspects of the interventions would yield the greatest benefit for future translation on a larger scale; the effects of the multicomponent intervention and bright light therapy on sleep of these patients as well as their effect on antipsychotic and sedative-hypnotic use.

## Methods/Design

### Project overview

The GMU concept was developed via an evidence-based approach using specific interventions that have been found to be beneficial for delirium care. GMU incorporated specific measures from the following programs: (i) Delirium Room which provides comprehensive medical care with multidisciplinary team meetings, initial behavioural and appropriate non-pharmacological methods being first line management in these patients [22] (ii) Concept of structured core interventions in Hospital Elder Life Program (HELP) [26-30] (iii) Use of bright light therapy to establish healthy sleep-wake cycle with appropriate timing to effectively shift altered circadian sleep-wake cycle to desired phase. In elderly patients with advanced sleep phase syndrome (ASPS), it has been found that evening exposure to bright light on daily basis can be beneficial [31-37]. This can be done with either a bright light box 1000-3000lux or spending time in outdoor sunlight 1-2 hours daily during late afternoon and evening.

The development of the GMU program included key considerations for room design and resource planning, program specific interventions and daily core interventions.

#### (1) Room design and clinical considerations in resource planning

Room design incorporated the aggregation of patients with delirium and difficult to control behaviours in a single 5-bedded cubicle. In Singapore, a multi-bedded system is common and hence would not cause additional concerns to patients and caregivers. Specific elder-friendly considerations include: 1) card access door; 2) low hospital beds; 3) individual night light; 4) falls modification in the toilet with movement-activated sensor lighting; 5) large wall clock; 6) calendars and 7) headboards indicating sensory impairments (if present), patient's favourite hobbies or activities to aid in individualised activity sessions. Other features of the GMU room include adequate lighting, panic button to ensure staff safety (in event of unpredictable combative patient behaviour) and a central space for group activities.

We aimed to provide higher nursing ratio of 1 trained nursing staff to 2.5 patients. The trained nurses, without distraction from other nursing and patient issues, are able to devote full attention to monitor the conditions of the 5 patients in the GMU and thereby able to initiate early interventions should they detect any deterioration in condition. This form of set up will ensure the reduction of the likelihood of injuries, falls and also allow active and early intervention to prevent functional decline and deterioration of the condition.

#### (2) GMU specific program and core interventions

The following program interventions were implemented by the multi-disciplinary team:

(1) Family orientation upon patients entering the GMU (with a standardised information sheet translated in all 4 languages with regards to the aims and interventions in the GMU). Family members are encouraged to visit patients daily to encourage communication and social support to the patients (Daily Visitor program).

(2) Geriatric nursing assessment and interventions (specific nursing assessment and interventions for cognitive and functional impairment, dehydration, nutrition, psychoactive medication use and discharge planning).

(3) The nurses involved in this program underwent a series of in-service training sessions with regards to the interventions including training on administration of cognitive screening tests and delirium scales; non-pharmacological and pharmacological management of delirium; delirium care and one-on-one interactions; communication skills in counseling concerned family members; psychogeriatrician teaching stress management skills; with support from the geriatric ward level nursing managers as well as cognition nurse clinicians who will counsel and aid in on-site difficult-to-manage behaviours. A pre- and post-implementation questionnaire of delirium knowledge was administered (table 2).

**Table 2:** Delirium knowledge questionnaire administered to staff of geriatric ward pre- and post- GMU implementation

	Knowledge Questions
1	Physical and/or chemical restraints used on a patient with delirium will protect them from harming themselves.
2	Patients with delirium will always present with agitation or restlessness.
3	We would be able to identify if the patient has delirium by administering the Abbreviated Mental Status Test (AMT).
4	Light therapy is beneficial in delirium management
5	Reality orientation can help in re-orientating the patient with delirium.
6	Dementia is the long term complication of delirium.
7	The first line management when a patient is unable to sleep is the use of a benzodiazepine (such as diazepam, lorazepam).
8	Delirium usually goes away immediately after the medical treatment is given.
9	Ensuring that the patient is well hydrated is an important factor in reducing delirium.
10	Confusion and delirium is part of normal aging.
11	The elderly patient may present with atypical symptoms that complicate the diagnosis of delirium.
12	Patients with functional decline will not benefit from early rehabilitation.

(4) Reference resources and practical reminders were made available for the staff in GMU.

(5) A continuing education program for care providers was implemented where the staff (doctors, allied health staff) involved in the GMU has regular training to increase awareness and knowledge in diagnosis and management of delirious patients.

(6) Nursing leaders helped support the trained nurses during the implementation stage.

(7) Daily geriatrician input with regards to these patients with daily documentation of mental status (Abbreviated Mental Status Test (AMT)/Chinese Mini Mental State Examination (CMMSE)) [37,38], Confusion Assessment Method (CAM) questionnaire [39] and type and severity of delirium (hyper-, hypo- or mixed delirium), Delirium Rating Scale (DRS) - severity [40], type of restraint use (physical and chemical including documentation of indication for restraint), pain assessment and medication management.

(8) Twice-weekly multidisciplinary rounds (involving the geriatricians, cognition nurse clinician, nurses, physiotherapists, occupational therapists, case coordinator, pharmacist, medical social worker, and dietician).

(9) Monthly meetings with the multidisciplinary staff members to evaluate the ongoing program, refine, voice out problems

(10) Early involvement of care co-ordinator or medical social worker (where appropriate) in discharge planning.

GMU core interventions are performed daily (see Table 3) and incorporated via a structured protocol into the daily nursing workflow and documentation sheet.

**Table 3:** GMU Core interventions

1.	No mechanical restraints and where possible, no pharmacological restraints. After trying all non-pharmacological methods and patient proves to be a danger to himself and others, then antipsychotics and sedative-hypnotics are used carefully at the lowest possible dose and to tail down the dose and remove the pharmacological agent once not required.
2.	Thrice daily patient orientation via reality orientation board
3.	Early mobilization with the help of therapists and trained nurses
4.	Provision of visual aids (such as eye glasses) if available
5.	Providing adequate hearing aids/earwax disimpaction where necessary with the use of portable audio amplifier
6.	Oral volume repletion/feeding assistance with scheduled oral intake schedule
7.	Sleep enhancement using non-pharmacological sleep protocol of warm milk, relaxation tapes or music. Sedative-hypnotic agents will again be the last line management.
8.	Bright light therapy from 6-10 pm
9.	Thrice daily therapeutic activities program for cognitive stimulation and socialization
10.	Minimizing immobilizing equipments like intravenous drip, urinary catheter, oxygen tubing
11.	Daily visitor program by family to encourage communication and social support
12.	Pain management

*These core interventions was then developed as standardized protocol and incorporated into the usual nursing assessment and daily documentation sheet by way of a checklist.

#### (3) Bright light therapy

Considerations in GMU design also included provision of a controlled environment with bright light therapy built into the room lights. Contributing factors to exacerbating delirium are changing physical environment and unfamiliarity of staff to the needs of delirious patients. The GMU is able to provide a controlled setting with minimal changes to the environment and nurses attending to them. In addition, bright light therapy will be carried out in this room from 6 pm to 10 pm (brightness of approximately 2000 to 3000 lux). The aim of bright light therapy is to establish healthy sleep-wake cycle with appropriate timing set to a desired phase. Since sleep deprivation is one of the contributing factors to worsening delirium, it will be beneficial to place such patients in this room to modulate their sleep-wake cycle. The peaceful environment, free from the movement and noises from other patients will allow better and uninterrupted period of sleep.

##### Inclusion, exclusion criteria and patient transfers

The admission criteria for GMU included patients above age of 65 years old assessed to have delirium (either on admission or incident delirium during hospital stay) who were admitted to the geriatric medicine department. Patients were excluded if they had medical illnesses which require special monitoring (e.g. telemetry for arrhythmias or acute myocardial infarction); were deemed to be dangerously ill, in coma or had terminal illness; uncommunicative patients or patients with severe aphasia; severely combative behaviour with high risk of harm; and patients with mania or other severe eye disorders and other contraindications to bright light therapy (such as patients on photosensitising medications). Finally we also excluded patients with respiratory or contact precautions, or there was verbal refusal of GMU stay by family/patient/physician-in-charge.

The GMU will have a dynamic system for transfers (akin to an intensive care unit) whereby patient flow is effected once they are assessed to have met entry or discharge criteria. This will be based upon both subjective and objective approach when the medical team deems the patients to have easy to manage behaviour/minimal or stable behavioural medications and objectively supported by decreased severity of delirium, based on DRS and CAM-severity scores.

##### Study design and patients recruitment

**Figure 1:**
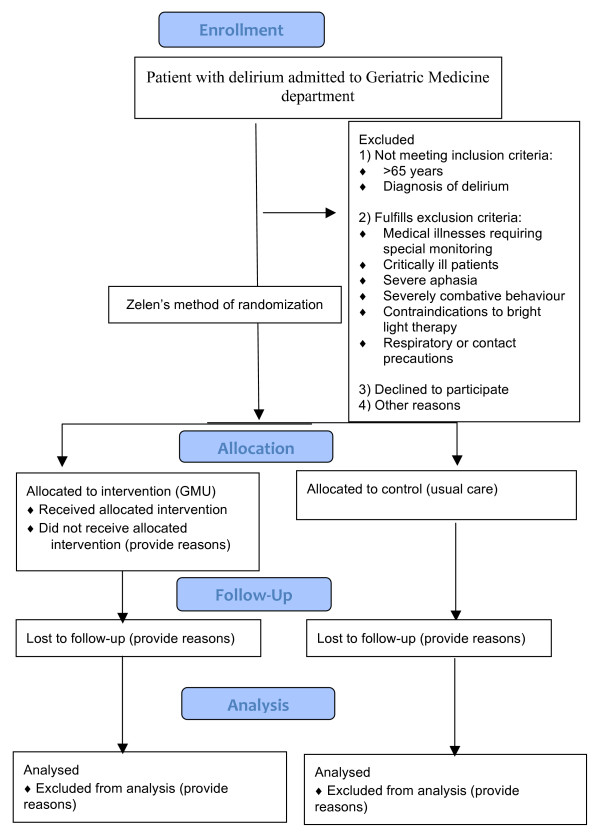
**Study design and subject allocation**.

Our study consist of a pre/post GMU implementation as well as concurrent GMU patients versus patients with delirium managed in the geriatric ward as controls. We will collect available data for patients with delirium admitted to the geriatric department one month prior to GMU implementation. We will give a one-month run-in period after establishment of GMU to smoothen out the initial teething issues and operational issues. The study recruitment commenced in November 2011. We will subsequently identify patients with delirium who meet the inclusion and exclusion criteria for GMU. Using Zelen's method of randomization [41,42], we will admit the patients to GMU and assign others to the control group (see Figure 1). The geriatric team managing the patients in both groups will speak with family regarding the study and if agreeable, consent with be obtained by the GMU co-ordinator and/or geriatric team.

##### Data collection

Table 4 summarizes the parameters and outcomes of interest. Data that will be collected include patient demographics (age, gender, race, length of hospital stay, duration of delirium(days); comorbidity and severity of illness data using modified Charlson's comorbidity index [43] and modified severity of Illness index [44]. Cognitive status will be assessed by way of the locally validated cognitive screening instrument (Abbreviated Mental Status Test (AMT)) [37] and functional status (using modified Barthel Index) [45] during the initial and pre-discharge phase of patient's admission. This was administered by a single trained assessor. We also aim to look at the rate and duration of physical restraint and frequency of chemical restraint use. To adjust for the differences antipsychotic usage, we will use chlorpromazine equivalence [46] to look at the total antipsychotic usage during the admission, as well as frequency of antidepressant and benzodiazepine use. Rate of falls and physical injuries, urinary catheter usage, complications of immobility (pressure ulcer) and rate of nosocomial infections will be systematically collected during patient's hospital stay. The functional outcomes in the geriatric patients with delirium include follow-up phone interview at baseline, 6 months and 12 months later. Hospitalisation, morbidity and mortality data over the same follow-up period will also be collected during those time intervals. These data will be obtained from patients in the GMU patients and usual care (control group). We will assess family/caregiver satisfaction via surveys conducted for loved ones of delirious patients in GMU versus those whom are receiving usual care.

**Table 4:** Overview of assessments used in GMU implementation study

Domain	Type of assessment/Outcomes			
		Pre-GMU implementation	GMU implementation	GMU concurrent controls
**Baseline demographics**	**Age, race, gender, length of hospital stay**	X	X	X
**Cognition**	**AMT** **CMMSE**	X -	X X	X X
**Comorbidity**	**Charlson's index** **SIRS**	X X	X X	X X
**Delirium severity**	**CAM severity score** **DRS-98**	- -	X X	X X
**Functional status**	**Modified BI**	X	X	X
**Physical restraints**	**Daily records**	X	X	X
**Medications**	**Antipsychotics (chlorpromazine equivalence)** **Antidepressants** **Benzodiazepines**	X	X	X
**Morbidity and mortality**	**Rate of falls** **Physical injuries** **Urinary catheter usage** **Pressure ulcer development** **Rate of nosocomial infections** **Inpatient mortality**	X	X	X
**Qualitative measures**	**Family satisfaction survey** **Staff satisfaction survey** **Staff knowledge survey**	-	X	X
**Follow-up data (6, 12-monthly)**	**Rate of hospitalization** **Functional status (BI)** **Institutionalisation** **Mortality**	-	X	X

The primary outcome measures we will look at include: (i) Clinical data with regards to duration and severity of delirium; frequency of use of physical restraints, use of pharmacological agents/sedative hypnotics, physical injuries or monthly falls rates; and nosocomial infection rates (ii) Functional outcomes by way of modified Barthel Index score and (iii) Patient/family satisfaction score; all adjusted for baseline demographics and comorbidities. We aim to look at the outcomes measures of patients with delirium admitted to GMU versus those managed under usual care (control group)(refer to Figure 1).

##### Sample size and statistical analyses

We aim to recruit 50 subjects during the pre-implementation and 50 subjects each from the GMU group and control (usual care) group (total 150 study subjects). We based our calculations on a two-sample comparison of means for the GMU and control group using non-repeated measures. Using mean Barthel Index score difference of 10 with standard deviation of 14 with ratio between groups of 1, a sample size of 45 patients in each group will be adequately powered to detect a 10-point mean Barthel Index group differences, with 90% power. Assuming that 10% of patients are lost to follow-up at 1 year, we project a final sample size of 50 (rounded up). Level of significance was set at 5%. Sample size calculation was performed in PS Power and Sample Size Calculations V2.1.30. Statistical analysis will be performed using appropriate statistical methods (univariate and multivariate analyses) for between group comparisons. Appropriate statistical methods will be used for the longitudinal outcome analyses and statistical significance is set at 5%.

##### Consent and ethical considerations

Informed consent for data collection will be obtained from the legal guardians of patients with delirium. Ethics approval has been obtained from National Healthcare Group Domain Specific Review Board (NHG DSRB).

## Discussion

We describe the rationale and concepts for GMU, a specialized 5- bedded unit dedicated to delirium care. Studies have shown that good leadership and effective management play a key role in bringing about a successful change to a positive workplace culture through innovative programs and research projects. Organisational investment can improve outcomes for staff stability and productivity, care quality and budgets, and better prepare those involved in the care of the elderly. The above provided the rationale for our development of a specialised delirium unit. The unit uses evidence-based practices of Delirium Room, HELP program and bright light therapy as its ground model.

The Delirium Room described by Flaherty et al is a specialized 4-bed unit that provides 24-hour intensive nursing care with a restraint-free concept [22]. This Delirium Room model provided comprehensive medical care, akin to that provided in the Acute Care of the Elderly (ACE) unit with multidisciplinary team meetings and initial behavioural and appropriate non-pharmacological methods being first line management in these patients. Low-dose antipsychotics or benzodiazepines were used judiciously (only when necessary basis), and avoided or discontinued whenever possible. Nursing staff was also trained with a constant sitter to the 4 patients. The study reported no difference in actual length of stay and expected length of stay for the Delirium Room patients. However, only 13% of subjects lost function while in the hospital. They also had low (29%) use of pharmacological restraints and only 8.7% used sedative-hypnotic agents.

While Delirium Room has been shown in literature to improve delirium care, the HELP program is primarily a multi-component intervention developed to prevent delirium in hospitalized older patients [26,27]. It has shown significant reductions in the number and duration of episodes of delirium in hospitalized older patients and involves standardized protocols in the management of the six major risk factors for delirium: cognitive impairment, sleep deprivation, immobility, visual impairment, hearing impairment and dehydration. We had incorporated this in the GMU in view of the structured nature of the core interventions administered in a systematised manner and that these principles were also reflective of good geriatric delirium management. This was further supported by delirium patient data, which showed complications of immobility (pressure ulcer rate of 44.7%). Our initial staff survey indicated that while staff members knew the core interventions help in delirium care, they were not translated into clinical practice. The novelty of this GMU model lies in the extrapolation of core interventions in the HELP program originally used for delirium prevention for actual management of delirium. While there are robust data to support the HELP program in delirium prevention, this has not been explored in the management of established delirium. In addition, we had also chosen specific multi-faceted intervention incorporating room design, core interventions in HELP program and bright light therapy. The role of bright light therapy in delirium care has previously only been published in the intensive care and post-operative settings [47,48].

While the GMU model has been developed for delirium care as a limited 5-bedded unit, it is unclear currently whether the benefits would be best seen in the hypoactive, mixed or hyperactive delirium. We had excluded combative hyperactive delirium patients who might cause the most caregiver/staff distress and who might require the most care time. However, this is done taking into account staff safety as well as safety of other patients. Also the combative delirious patient might least benefit from the core interventions as he/she might not be able to co-operate with the respective strategies applied. In order to look at the subtype of delirium patients who might best benefit from the GMU, we will further review their delirium and functional outcomes post-GMU admission to refine and improve our model in the future. We hope that the development of GMU will improve both short and long-term outcomes of hospitalized patients with delirium compared to usual care. This model of care in future may also be translated in a trans-disciplinary manner across other medical and surgical wards (akin to ACE unit model) where delirious patients could be sited.

## Competing interests

The authors declare that they have no competing interests.

## Authors' contributions

CMS, MC participated in the design of the GMU and writing up of manuscript. JK and HHC participated in the design of GMU, design and administration of satisfaction questionnaire and design of structured GMU nursing protocol. DYY and TTL participated in design of GMU and inputs into the manuscript. All the authors read and approved the final manuscript.

## Pre-publication history

The pre-publication history for this paper can be accessed here:

http://www.biomedcentral.com/1471-2318/11/41/prepub
